# Simultaneous aortic and pulmonary valve replacement in a young patient after intracardiac repair for tetralogy of Fallot: mechanical or biological valve? A case report

**DOI:** 10.1093/jscr/rjab170

**Published:** 2021-05-17

**Authors:** Ryo Okubo, Fumiaki Kimura, Hideyuki Harada, Daita Kobayashi, Tomonori Shirasaka, Hiroyuki Kamiya

**Affiliations:** Department of Cardiac Surgery, Asahikawa Medical University, Asahikawa, Japan; Department of Cardiovascular Surgery, Kushiro Kojinkai Memorial Hospital, Kushiro, Japan; Department of Cardiovascular Surgery, Kushiro Kojinkai Memorial Hospital, Kushiro, Japan; Department of Cardiovascular Surgery, Steel Memorial Muroran Hospital, Muroran, Japan; Department of Cardiac Surgery, Asahikawa Medical University, Asahikawa, Japan; Department of Cardiac Surgery, Asahikawa Medical University, Asahikawa, Japan

## Abstract

Pulmonary valve stenosis and regurgitation can occur in the distant stages after intracardiac repair of tetralogy of Fallot (TOF). Aortic regurgitation (AR) can also occur, although it is rare in postoperative patients. However, there are few reports of simultaneous replacement of the pulmonary and aortic valves in young patients after intracardiac repair of TOF, and there are no clear guidelines for selecting a valve prosthesis in such patients. We report a case of severe pulmonary valve stenosis and regurgitation with severe AR 38 years after the TOF operation, in which urgent double valve replacement and right ventricular outflow tract patching were performed with a mechanical valve in the aortic valve position and a bioprosthetic valve in the pulmonary valve position, with a successful outcome.

## INTRODUCTION

Pulmonary valve stenosis and regurgitation can occur in the distant stages after intracardiac repair of tetralogy of Fallot (TOF). Aortic regurgitation (AR) can also occur, although it is rare in postoperative patients, and there have been reports of patients requiring aortic valve replacement after intracardiac repair of TOF [1]. However, there are few reports of simultaneous pulmonary and aortic valve replacement in young patients after intracardiac repair of TOF, and there are no clear guidelines for selecting a valve prosthesis in such patients.

Here, we report a case of severe pulmonary valve stenosis and regurgitation (PSR) with severe AR 38 years after the TOF operation, in which urgent double valve replacement and right ventricular outflow tract patching were performed with a mechanical aortic valve and a bioprosthetic pulmonary valve, with a successful outcome.

## CASE REPORT

The patient was a 42-year-old man who had undergone a radical operation for TOF at the age of 4 years, although the details are unknown. At the age of 41 years, he was admitted to our hospital for heart failure, and right heart catheterization revealed PSR. A myocardial biopsy showed no abnormalities, and he recovered with conservative treatment. However, he experienced recurrence of congestive heart failure, resulting in re-admission.

**Figure 1:**
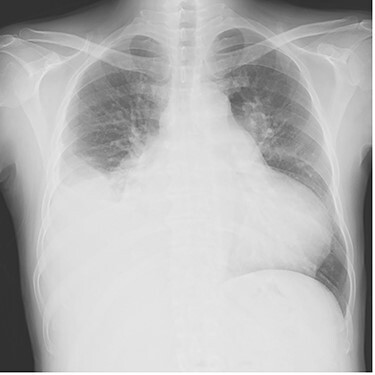
Plain chest radiograph at the time of admission.

Chest radiography showed an enlarged heart with a cardiothoracic ratio of 73% and right pleural effusion (Fig. 1). Laboratory test results were as follows: blood urea nitrogen, 48.1 mg/dl; creatinine, 1.96 mg/dl; total bilirubin, 2.7 mg/dl and N-terminal prohormone of brain natriuretic peptide: 3043 pg/ml. Transthoracic echocardiography revealed ejection fraction of 49%, severe AR, mild to moderate mitral regurgitation, severe pulmonary regurgitation and mild to moderate tricuspid regurgitation. The pulmonary valve’s maximum pressure gradient had decreased from 31 mmHg 8 months previously to 5 mmHg. The diameter of the ascending aorta was 39.2 mm, sinotubular junction was 36.1 mm and Valsalva sinus was 47.4 mm. Cardiac catheterization showed no significant coronary artery stenosis.

**Figure 2:**
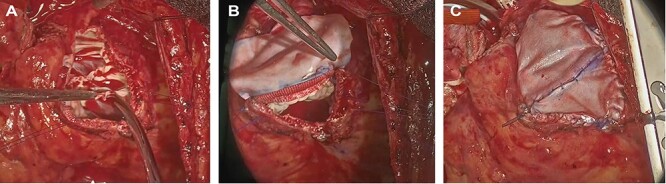
Intraoperative photograph; (**A**) The remnants of the pulmonary valve; (**B**, **C**). pulmonary valve replacement and patching.

Although the patient responded to conservative treatment for several days after admission, ventricular arrhythmia appeared on the 11th day and his general condition worsened rapidly. Therefore, we performed surgery on the 15th day.

The surgery was performed through median re-sternotomy, and cardiopulmonary bypass was established with arterial cannulation into the right femoral artery and venous cannulation into the right femoral vein and later into the superior vena cava. Cardiac arrest was induced with retrograde cold blood cardioplegia. A Dacron patch was seen from the right ventricular outflow tract to the main trunk of the pulmonary artery, and when an incision was made, a severely destroyed pulmonary valve was observed. Remnants of the pulmonary valve and the myocardium of the right ventricular outflow tract, which was stenotic, were removed (Fig. 2a), and pulmonary valve replacement and patching were performed using a bioprosthetic valve (Epic mitral 29 mm, St. Jude Medical, Inc., St Paul, MN) and bovine pericardium (Fig. 2b and c). The aortic valve was observed through an aortic incision. It was tricuspid, and the right coronary cusp was prolapsed. There was no vegetation suspicious for infective endocarditis. A mechanical valve (Regent 27 mm, St. Jude Medical, Inc., St Paul, MN) was used for aortic valve replacement. The minimum rectal temperature was 31.4°C, extracorporeal circulation time was 355 min, and aortic cross-clamp time was 184 min.

**Figure 3:**
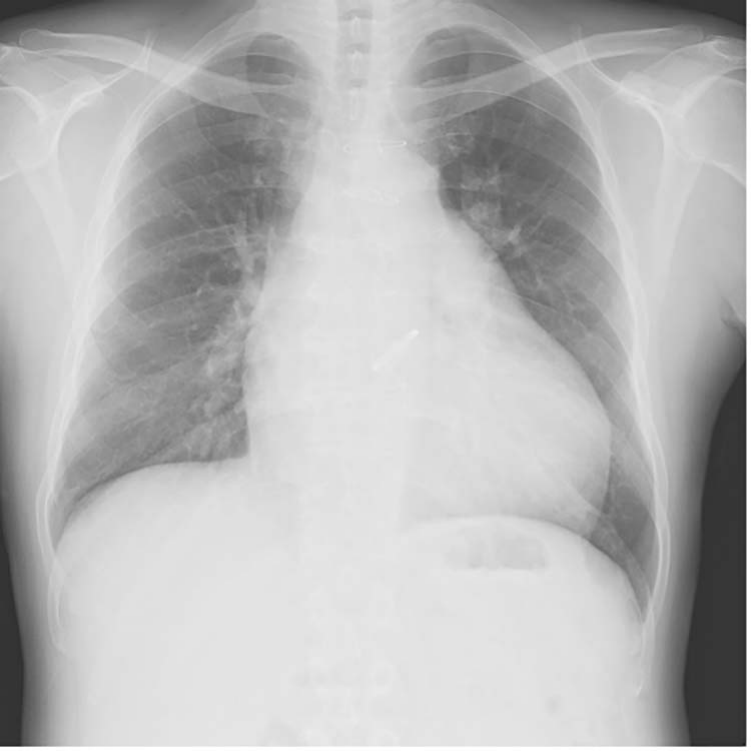
Plain chest radiograph of the first outpatient visit after discharge from the hospital.

The patient was extubated on the third postoperative day. He was weaned off catecholamines on postoperative Day 9 and transferred from the intensive care unit on postoperative Day 10. Postoperative transthoracic echocardiography revealed no prosthetic valve dysfunction at either the aortic or pulmonary positions. Although his general condition was good, he had prolonged wound healing in the right inguinal region and resisted conservative treatment. Patchplasty of the right common femoral artery was performed on postoperative Day 49. He was discharged on postoperative Day 62. A plain radiograph from the first post-discharge outpatient visit is shown in Fig. 3. Two years postoperatively, the patient was doing well without heart failure.

## DISCUSSION

We performed urgent two-valve replacement and patching of the right ventricular outflow tract in a patient with PSR and severe AR in the distant phase after TOF surgery. When simultaneous aortic and pulmonary valve replacement are required, there are no clear guidelines for choosing a prosthetic valve. Regarding aortic valve replacement, mechanical valves are recommended for patients younger than 50 years, considering the risk of valve dysfunction [2]. However, whether mechanical or biological valves should be used for the pulmonary position remains controversial.

In the pulmonary position, more aggressive anticoagulation is necessary because thrombosis is more prevalent in the right-sided position when mechanical valves are used [3]. However, biological valves have limited durability. Neukamm *et al.* reported a combined 5-year postoperative survival and valve event avoidance rate of 91% for pulmonary valve position bioprosthesis in 56 young patients, with good mid-term results [4]. Tokunaga *et al.* compared mechanical and bioprosthetic pulmonary valves in a small number of cases and reported no significant difference in the avoidance rate of valve-related events and more cases of thrombosis with mechanical valves [5]. In the present case, a mechanical valve was used for the aortic position and a biological valve for the pulmonary position to maintain an international normalized ratio of prothrombin time (PT-INR) between 2 and 2.5 for avoiding bleeding complications in the late follow-up period, because PT-INR should be between 3 and 3.5 when mechanical valves are used in the pulmonary position. At the time of surgery, we considered the possibility of future transcatheter pulmonary valve replacement, which is not yet approved in Japan. Webb *et al.* reported valve-in-valve transcatheter pulmonary valve replacement in six patients; this technique was considered suitable because of the low-pressure system and history of repeated surgery [6].

In conclusion, choosing prosthetic valves in the pulmonary and aortic positions in young patients is difficult and controversial; the decision should be made based on individual patients. However, considering the future development of transcatheter therapy, using a bioprosthetic valve in the pulmonary valve position could be considered to maintain low-level PT-INR and avoid reopening surgery.

## References

[1] Dodds GA 3rd, Warnes CA, Danielson GK. Aortic valve replacement after repair of pulmonary atresia and ventricular septal defect or tetralogy of Fallot. J Thorac Cardiovasc Surg 1997;113:736–41. 9104983 10.1016/S0022-5223(97)70232-0

[2] Taherkhani M, Hashemi SR, Hekmat M, Safi M, Taherkhani A, Movahed MR. Thrombolytic therapy for right-sided mechanical pulmonic and tricuspid valves: the largest survival analysis to date. Tex Heart Inst J 2015;42:543–7. 26664307 10.14503/THIJ-14-4659 PMC4665281

[3] Otto CM, Nishimura RA, Bonow RO, Carabello BA, Erwin JP 3rd, Gentile F, et al. 2020 ACC/AHA guideline for the management of patients with valvular heart disease: executive summary: a report of the American College of Cardiology/American Heart Association Joint Committee on clinical practice guidelines. Circulation 2021;143:e35–71. 33332149 10.1161/CIR.0000000000000932

[4] Neukamm C, Lindberg HL, Try K, Døhlen G, Norgård G. Pulmonary valve replacement with a bovine pericardial valve: a five year follow-up study. World J Pediatr Congenit Heart Surg 2014;5:534–40. 25324250 10.1177/2150135114542165

[5] Tokunaga S, Masuda M, Shiose A, Tomita Y, Morita S, Tominaga R. Isolated pulmonary valve replacement: analysis of 27 years of experiment. J Artif Organs 2008;11:130–3. 18836873 10.1007/s10047-008-0413-8

[6] Webb JG, Wood DA, Ye J, Gurvitch R, Masson JB, Rodés-Cabau J, et al. Transcatheter valve-in-valve implantation for failed bioprosthetic heart valves. Circulation 2010;121:1848–57. 20385927 10.1161/CIRCULATIONAHA.109.924613

